# Conservative Gynecology and Electro-Therapeutics

**Published:** 1899-07

**Authors:** 


### BOOK NOTICE.

CONSERVATIVE GYNECOLOGY AND ELECTRO-THERAPEUTICS. A practical treatise on the Diseases of Women and their Treatment by Electricity. By G. Betton Massey, M. D., Physician to the Gynecological Hospital, etc. Third edition, revised, rewritten, and greatly enlarged. Philadelphia, New York, and Chicago. The F. A. Davis Co. 1898.

This book has been reviewed in its earlier editions in this journal before.

The first edition was reviewed in the number for August, 1889, page 358, and the second edition in July, 1890, page 329.

The issue now under review is the third edition.

The author is well known to the editor of THE HOMŒOPATHIC PHYSICIAN, who can unhesitatingly say that he stands at the head of the profession in Philadelphia as an electrical specialist. He is in every sense a fully trained physician, paying, however, special attention to electrical methods of therapeutics. His book in this light has the value of being the highest authority on the subject of which it treats.

Those who have the previous editions will hardly recognize the present volume as a third edition so greatly has it been expanded.

Says the author in the preface, "What was a mere treatise on the use of electricity in fibroid tumors and certain other affections has been transformed into a treatise on the medical and surgical diseases of women with special reference to the therapeutic use of electricity. This broadening of the scope of the work has been made necessary by the fact that nearly all the modern treatises on gynecology have been written from a purely surgical standpoint."

The author distinguishes himself and commends himself to the favorable consideration of all people who are not riding a hobby, but whose minds are open to the consideration of all sides of a therapeutic problem, by his denunciation of the indiscriminate performance of operations upon the genital organs of women. Thus at page 11 he deprecates "the removal of an ovary for pain due, in reality, to lateral sclerosis of the spinal cord, and the removal of both ovaries for similar symptoms due to scoliosis of the spinal column." He also similarly denounces indiscriminate abdominal surgery, and says: "No organ should be subjected to a mutilating operation, certainly none removed from the body, *until the powers of conservative medication have been intelligently tested*." Electricity in the hands of our author *is* "conservative medication" and is most intelligently applied as a further examination of his pages will abundantly show.

He positively disclaims electricity as a cure-all, and does not wish the reader's mind so filled with ideas of electricity that he will neglect any simpler means of treatment.

For the treatment of tumors there is given the whole process of electrolysis, with the philosophy and with suggestive experiments to enable the student the better to understand the principles involved.

The principle of cataphoresis is very well explained and is one of the author's specialties in treatment, as we shall soon see. Cataphoresis may be explained in the words of the author, as follows: "There is another physical accompaniment of the transmission of galvanic currents through a liquid-containing conductor such as the body, and that is the actual transfer of liquids and solids (in solution or in small particles) through it in the direction of the current. This is analogous to ordinary osmosis, but is entirely determined in direction and amount by the current. It is called catophoresis, and since medicaments can be inserted into the body by it, chiefly from the anode or positive pole, it has also been termed 'anodal diffusion.'"

The process is defined by the author very completely, the quotation here made being only a part of the whole statement.

The author has originated a most unique use for it. He applies it to the dissemination of Mercury in atomic proportions directly into diseased tissues. In this process the electricity is literally driven forward into the tissues.

Turning now from the definition of catophoresis on page 42 to page 236, we learn how the author uses cataphoresis in the treatment of cancer.

A gold needle electrode is amalgamated with Mercury and made the positive pole of a powerful galvanic battery. This pole is then inserted into the cancerous tumor while the negative pole, consisting of a wet pad, is applied to the outer skin. Under the influences of the electrical current flowing off from the positive pole into the tumor, the Mercury is carried into the tumor and there produces a "lethal effect" upon the tumor by which it is made to disappear. The author then quotes a number of cases treated with more or less success. He is, as usual, very conservative in his claims, but points out that it is a suggestion of what may yet be done in this direction.

This idea of treating cancerous tumors is the most important point in the whole book and the one to which the attention of readers of this journal is particularly directed. As the author well says of his methods that he has obtained "results, which though few in number and not always successful, yet point to the germ of a truth of vast importance to the human race."

It is to be distinctly understood that the author of the book is the sole originator of this method. On that point he says: "The underlying principle that constitutes the novelty of the method is that there is a virtue in the electrical diffusion of nascent chemicals throughout a malignant growth, which when of sufficient density per area will cause an interstitial death and ultimate absorption of the malignant cells at a distance from the electrode, without destroying the connective tissue surrounding them, and that this intracellular lethal action is independent of and additional to the ordinary destructive action of a strong current in the immediate neighborhood of the electrodes."

There is much more than the above points considered in this excellent book, but time and space are wanting. The book is embellished with an immense number of cuts and many colored plates. The plates representing the appearance of the os uteri in life while suffering from leucorrhœa are the best we have even seen.

Details of apparatus, minute directions for manipulating, diagrams for explaining principle of action of the various apparatus, and tables showing details of treatment and results in treating a great number of fibrous tumors, enrich the book and make it indispensable to every practitioner who seeks to make use of electricity.

